# Innate immune functions of avian intestinal epithelial cells: Response to bacterial stimuli and localization of responding cells in the developing avian digestive tract

**DOI:** 10.1371/journal.pone.0200393

**Published:** 2018-07-06

**Authors:** Enav Bar Shira, Aharon Friedman

**Affiliations:** Department of Animal Sciences, Robert H. Smith Faculty of Agriculture Food and Environment, The Hebrew University of Jerusalem, Rehovot, Israel; Universitat Hohenheim, GERMANY

## Abstract

Intestinal epithelial cells are multi-tasked cells that participate in digestion and absorption as well as in protection of the digestive tract. While information on the physiology and immune functions of intestinal epithelial cells in mammals is abundant, little is known of their immune function in birds and other species. Our main objectives were to study the development of anti-bacterial innate immune functions in the rapidly developing gut of the pre- and post-hatch chick and to determine the functional diversity of epithelial cells. After establishing primary intestinal epithelial cell cultures, we demonstrated their capacity to uptake and process bacteria. The response to bacterial products, LPS and LTA, induced expression of pro-inflammatory cytokine genes (IL-6, IL-18) as well as the expression of the acute phase proteins avidin, lysozyme and the secretory component derived from the polymeric immunoglobulin receptor. These proteins were then localized in gut sections, and the goblet cell was shown to store avidin, lysozyme as well as secretory component. Lysozyme staining was also located in a novel rod-shaped intestinal cell, situated at different loci along the villus, thus deviating from the classical Paneth cell in the mammal, that is restricted to crypts. Thus, in the chicken, the intestinal epithelium, and particularly goblet cells, are committed to innate immune protection. The unique role of the goblet cell in chicken intestinal immunity, as well as the unique distribution of lysozyme-positive cells highlight alternative solutions of gut protection in the bird.

## Introduction

Intestinal epithelial cells (IEC) form a highly organized cellular system, which is maintained in a dynamic steady state by proliferating and differentiating cells, and that is constantly renewed by multipotent stem cells originating in the crypts of Lieberkühn, located at the base of the intestinal villi [1]. In mammals, these stem cells give rise to four predominate epithelial lineages: absorptive enterocytes, goblet cells, entero-endocrine cells and Paneth cells.

IEC are multi-tasked cells that participate in digestion and absorption as well as in protection [2]. Protective properties of IEC in the mammal include formation of the gut barrier by enterocytes, mucin secretion by goblet cells, and antimicrobial protein secretion by Paneth cells; in fact, all IEC have been shown to express and secrete pro-inflammatory cytokines and chemokines. Thus, IEC actively defend the epithelial surface, and aid in recruiting immune cells [3]. The cross talk between IEC and other cell types found in the intestinal milieu is important for maintaining homeostasis, and involves growth factors, cytokines (such as IL-6, IL-1β), and chemokines (such as CCL20 [MIP3α] and CXCL8 [IL-8]), as well as ECM proteins [4]. Thus, additional to their tasks in nutrition IEC may also be viewed as fully fledged innate immune cells [2].

The ability of IECs to recognize and respond to multiple microorganisms relies upon a set of receptors that recognize conserved bacterial and viral motifs. These include lectins and adhesins, the nucleotide-binding oligomerization domain (NOD) family, and the Toll-like receptors (TLRs) which collectively function as pattern recognition receptors (PRRs) [2]. Cytokine and chemokine secretion collectively recruit leukocytes to the intestine, facilitate antigen presentation to immune cells, and enterocytes may function as antigen presenting cells and regulate lymphocyte responses in the intestine [5, 6]. Recent findings show that intestinal goblet cells are capable of sensing microbiota, actively sample bacteria and then transfer them to underlying CD103^+^ dendritic cells which imprint gut homing on lymphocytes, promote IgA production and induce development of regulatory T cells [7–9].

In contrast to the vast and growing information on IEC physiology and immune functions in mammals, almost nothing is known of their immune function in other species. Despite the lack of information, studies suggest that mucosal epithelial cells of birds, similar to mammals, actively participate in innate immune responses against harmful pathogens. Eren *et al* showed that IEC of day old chicks express both TLR4 and TLR2 [10], and additional studies showed that avian mucosal epithelial cells, including enterocytes, produce and secrete various antibacterial compounds which include lysozymes,β defensins, cathelicidins and avidin [11–16]. Moreover, it was shown that avian enterocytes obtained from orally BSA-immunized birds responded in an antigen specific manner to additional antigenic stimulus, and were suggested to be active participants in induction of intestinal anaphylaxis [17, 18]. In our studies, we have demonstrated the importance of avian intestinal goblet cells in preserving maternal IgA antibodies, thus preserving maternal protection invested by the hen for the newly hatched chick [19].

The development and function of IEC in precocial birds (the domestic fowl) is of particular interest, as a dramatic switch from the embryonic digestive tract to a fully functional adult digestive tract occurs within the first 24 hours after hatch. While this dramatic change has been investigated in terms of digestion, absorption and microbiome colonization, its implications on the development of immune functions in the avian gut have yet to be fully appreciated. Furthermore, the precise types, roles and location (in the villi and throughout the gut) of avian IEC remain ambiguous. As the immune protection of the pre- and post-hatch chick depends on both maternal as well as innate immunity [19, 20], our main objectives herein were to establish the development of anti-bacterial innate immune functions in the rapidly developing gut of the pre- and post-hatch chick. Additionally, we investigated the functional diversity of IEC along the digestive tract in chick embryos and hatchlings.

## Materials and methods

### Animals and husbandry

Fertile White Leghorn eggs were purchased from a commercial hatchery (E.M.I. Mesout Izchak, Israel) and incubated on a wire mesh in a semi-automated incubator. Embryos from these eggs were used for selected experiments. After hatching, chicks were placed in floor pens on wood shavings in isolated, disease-free, light and temperature-controlled rooms at 32°C for the first week post-hatch, followed by 28°C during the second week. The feed was a commercial starter formulated to meet or exceed NRC requirements (Matmir Feed Co., Beit Shemesh, Israel). Feed and water were ascertained to be pathogen-free and were provided ad libitum for the entire experimental period. All experimental procedures were approved by the Animal Ethics and Welfare Committee of the Authority for Biological and Biomedical models, the Hebrew University of Jerusalem (Ethics committee research number: AG-12-13298-2; NIH approval number: OPRR-A01-5011).

### Isolation of IEC for in vitro studies

Chicks and chicken embryos were euthanized by cervical dislocation on different days pre- and post-hatch (*n* = 5 for each time point). Intestinal segments were identified and removed. Epithelial cells were isolated using a modification of the technique described by Meddings et.al. [21], as previously detailed [19]. The segment washes were pooled, and IEC were collected from the wash-out by gentle centrifugation at 300xg. Pooled IEC were then subjected to RNA extraction.

Following extraction of IEC, segment samples from the small intestine were collected, and immediately fixed in buffer formaldehyde solution (Frutarom, Acre, Israel) for at least 24 h at room temperature. The samples were then dehydrated, embedded in paraffin (Thermo Shandon, Pittsburgh, PA, USA), sectioned (4μm), and stained with hematoxylin and eosin (Thermo Shandon, Pittsburgh, PA, USA). Histological slides were examined for confirmation of appropriate isolation of the epithelial layer leaving intact lamina propria (not shown). Additionally, the isolated IEC were examined for the expression of villin as a marker for intestinal epithelium [22].

### Primary epithelial cell culture

Whole intestines were removed from E17 chick embryos into DMEM-F12 medium (Sigma- Aldrich Company, Ayrshire. UK). Individual Intestines were washed and segmented into duodenum (pancreatic loop), cecum (two ceca were disconnected from the intestines at the ileo-cecal junction) and colon. Each segment was then added to a pool of identical segments. The pancreas was removed from duodenal loops, and loop segments were split lengthwise and sliced using a sterile scalpel blade. Slices were forced thru a stainless steel mesh (55mm diameter 100 micron opening, Sigma-Aldrich, Rehovot IL). The resulting fragments were collected, washed and suspended in complete DMEM-F12 medium containing: penicillin 100 U/ml, streptomycin 0.1 mg/ml, nystatin 12.5 U/ml, sodium pyruvate 0.11 mg/ml (all from Biological Industries, Beit Haemek, Israel), Glutamax™ 2 mM (Thermo-Fisher Scientific, Carlsbad, CA, USA), and BD™ Mito-serum Extender (BD Biosciences, Bedford, MA, USA. The resulting suspension was seeded into six well plates (Nunc), previously thinly coated with BD Matrigel matrix (Erembodegem, Belgium). The same procedure was applied to segments derived from ceca and colon. Plates were incubated at 37.5°C, 7.5% CO_2_. Under these conditions, epithelial monolayers develop within 48h.

### RNA extraction and PCR analysis

RNA was extracted from IEC using RNAzol™ (Molecular Research Center Inc.) according to the manufacturer’s instructions. Contaminating chromosomal DNA was digested with DNase I (RNAse free; 1 U/μg of RNA; Thermo Scientific, Fermentas Molecular Biology Tools) for 20 min at 37°C. RNA quality was assessed using Agilent Bioanalyzer. 0.5–1μg RNA from each sample was reverse transcribed using iScript^™^ Advanced cDNA Synthesis Kit for RT-qPCR (Bio–Rad) according to the manufacturers’ protocols. cDNA was amplified by PCR using SsoFast^™^EvaGreen® Supermix (Bio-Rad) and specific primers for the examined genes (Table 1). Primer sequences were designed according to GeneBank published sequences. Each primer pair was calibrated to determine the optimal reaction temperature and cDNA concentration.

**Table 1:** PCR primer pairs.

Primer Pair	Gene Bank
Sense 5’-AAGGGTGGTGCTAAGCGTG-3’	GAPDH
Antisense 5’-ATGGCATGGACAGTGGTCATAA-3’	NM_204305.1
Sense 5’-CGGCGTCCAACTTCTTAGAGG-3’	18S
Antisense 5’-CTGCCGGCGTAGGGTAGACAC-3’	AF173612
Sense 5’-TCAAGGTGCCACATCCATAC-3’	TLR4
Antisense 5’-CTCCTGCAGGGTATTCAAGT-3’	NM_001030693.1
Sense 5’-ACATGATCTGCAAAAGGC-3’	TLR2
Antisense 5’-TGAATGCGAAGGTGTTGG-3’	NM_204278
Sense 5’-CACCTTTGGCTTCACCGTG-3’	Avidin
Antisense 5’-TGTTGATGCCGACCCT-3’	NM_205320.1
Sense 5’-ACATAACAGCGAGCGTGAAC-3’	Lysozyme
Antisense 5’-CTCCTCACAGCCGGCAGCCT-3’	NM_205281
Sense 5’-TCGAAAGAGTGGCTTCTGTG-3’	AVBD1
Antisense 5’-AGGTCAATGGGGGGAAGTTTC-3’	NM_204993.1
Sense 5’-AGAAATCCCTCCTCGCCAAT-3’	IL6
Antisense 5’-AAATAGCGAACGGCCCTCA-3’	NM_204628.1
Sense 5’-AGGTGAAATCTGGCAGTGGAA-3’	IL18
Antisense 5’-TATCTTCTACCTGGACGCTGA-3’	NM_204608
Sense 5’-GACTTCCCCATCCTCATCCG-3’	CD14
Antisense 5’-CACACCTTGCCTTTCACAATGTTC-3’	NM_001139478.1
Sense 5’-AAGATGCTGGCCTGTACCT-3’	GGp-IgR
Antisense5’-CGGGTCGTAGTGGCAATCAAT-3’	NM_001044644.1
Sense 5’-TAACCTGGGCGATGTCTTCC-3’	Villin
Antisense 5’-CCACCCGCCAGACCTCT-3’	J03781

Expression levels of examined genes was determined using Real Time PCR using C1000 Thermal Cycler (Bio-Rad). Results were analyzed using Bio Rad CFX manager*™* software. Dissociation curve analysis was performed at the end of each real-time PCR reaction to validate the presence of a single reaction product and lack of primer dimerization. Expression levels of examined genes were normalized using at least 2 normalizing genes (GAPDH, 18S; Table 1).

### Histology and Immunohistochemistry

Tissue samples were obtained from chicken embryos or post-hatch chicks and immediately fixed overnight in 3.5% buffered paraformaldehyde solution (0.1M, pH 7.4) containing 0.1% gluteraldehyde (Frutarom, Acre, Israel) at 4°C. The samples were then dehydrated and embedded in paraffin (Thermo Shandon, Pittsburgh, PA, USA). For Immunohistochemistry, 1–3μm thick sections were stained with primary antibodies alone or with a combination of primary and secondary antibodies. Primary antibodies used were: mouse monoclonal anti-human secretory component (clone GA-1; Sigma-Aldrich, Inc., Israel), purified mouse or rabbit IgG anti-lysozyme prepared in our lab (Briefly, a highly purified source of hen egg white was used for immunization [L4631, Sigma-Aldrich, Israel]; the resulting antibodies did not cross react with avidin, ovalbumin or ovotransferrin), polyclonal rabbit anti-chicken avidin (Thermo-Fisher Scientific), polyclonal goat anti-Muc2 (R12) (Santa-Cruz Biotechnology, Inc., Dallas, TX, USA) Detecting (secondary) antibodies used were: HRP labeled goat anti-mouse IgG (H+L) (KPL Gaithesburg, MD, USA) or HRP labeled goat anti-rabbit IgG (H+L) (Jackson Immuno Research, Laboratories, Inc., West Grove, PA, USA) and Alexa Fluor® 488 donkey anti goat IgG (H+L) (Jackson Immuno Research, Laboratories, Inc., West Grove, PA, USA).

For immunofluorescence, we used a modified procedure previously described by Derache [23]. Fixed cultured epithelial cells were stained with antibodies previously shown to cross react with the respective chicken proteins [23], mouse anti-villin (Abcam, Cambridge UK) or mouse anti-E-cadherin (clone 36, BD Biosciences) antibodies, and then detected by Alexa Fluor 488-conjugated secondary antibody (Sigma-Aldrich). Cell and tissue slides were observed with the BX 51 fluorescent microscope (Olympus, Japan) fitted with a DP-72 camera. Image analysis and processing (tone adjustment, cropping and image sharpening) was performed with Adobe Photoshop CS6 software.

### Bacteria processing and stimulation with bacterial PAMPs

Cecal IEC obtained from E17 embryos were cultured as described above. Two days after epithelial culture initiation, medium was replaced with stimulatory medium containing pHrodo™ Green *Escherichia Coli* (*E*. *Coli*) BioParticles® or pHrodo™ Red *Staphylococcus aureus* (*S*. *aureus*) BioParticles® conjugates (Molecular probes, life technologies, Eugene, OR, USA). According to the manufacturer, these conjugates are non-fluorescent outside the cell at neutral pH but fluoresce brightly at acidic pH (such as found in phagosomes). The cultures were monitored hourly and when fluorescence developed micrographs were taken.

To test epithelial cell responses following stimulation with bacterial products a similar protocol was applied, but cells were stimulated with medium containing either *Salmonella typhimurium* LPS (a constituent of Gram negative bacterial outer membrane) (Sigma-Aldrich Co., St. Louis MI 1640) or *Bacillus subtilis* LTA (a major constituent of Gram positive bacterial cell wall) (Invivogen, San Diego, USA) at 1μg/ml or 10μg/ml. Stimulation was administered for 6 hours, after which the cultures were washed several times with medium and then viewed microscopically or subjected to RNA extraction. In control cultures, medium was replaced with fresh medium at the time of stimulation.

### Statistical analysis

Statistical analyses and graphs were performed using JMP® software (SAS® Institute Inc., Cary NC, USA) using one-way analysis of variance (ANOVA) to determine the significance of differences and the interactions between experimental groups and experiments. Data were analyzed using Tukey HSD test or Student's T test to determine significance of differences between group mean values; comparisons were made between the experimental groups and respective controls. Values were considered significantly different, in the least, at P<0.05.

## Results

**Fig 1:**
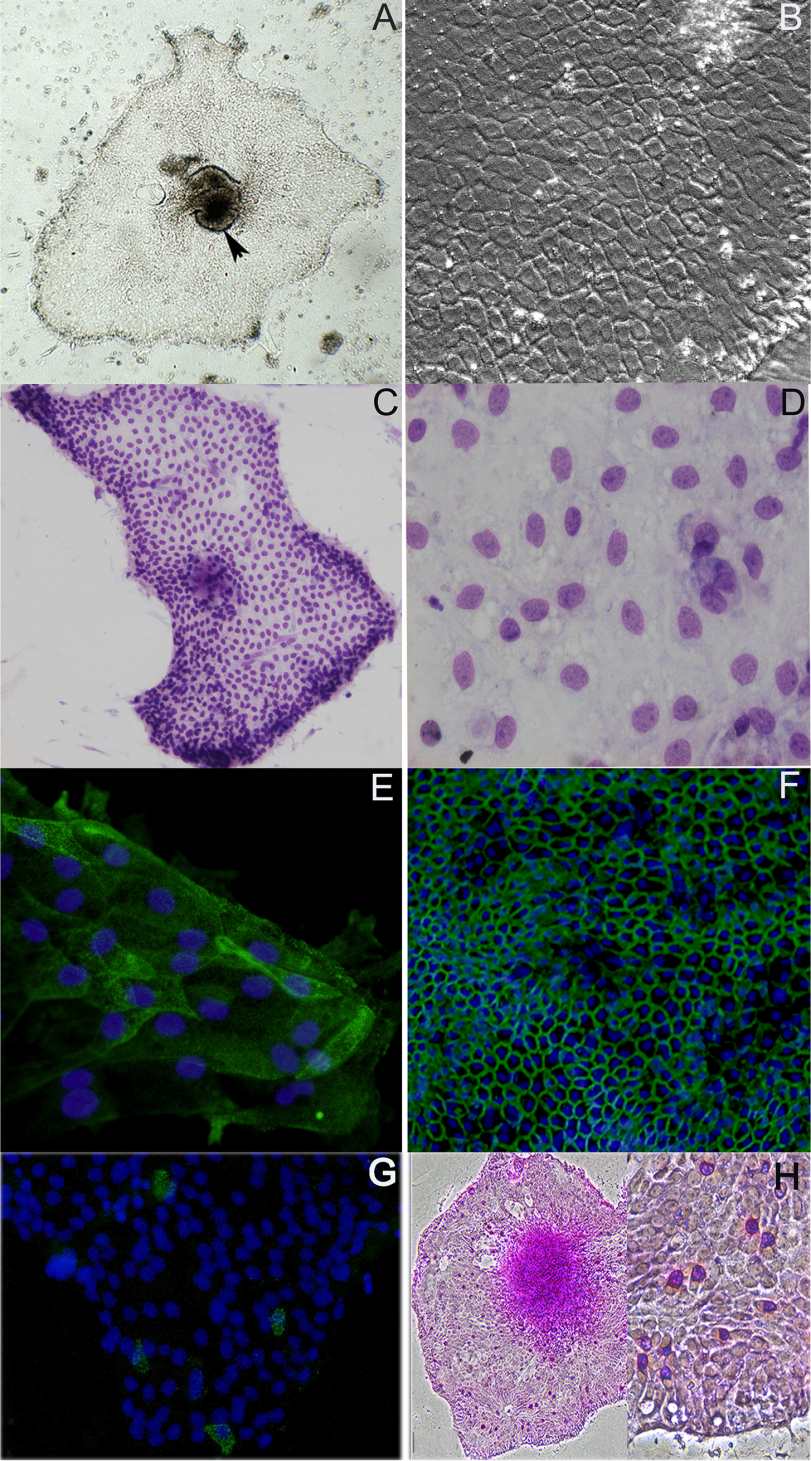
Morphological characterization of chick IEC cultures. Micrographs A & B show a typical epithelial monolayer under phase contrast microscopy. In A, the arrow head shows the intestinal fragment from which the monolayer originated. The monolayer gradually extended and assumed a typical mosaic structure (B) typical to epithelium. These structures are further characterized by light microscopy (May-Grunwald staining) (C & D). The typical mosaic structure is seen in the central area of D. The epithelium was stained with two FITC labeled polyclonal antibodies: mouse anti-villin antibody (E) and mouse anti-E-cadherin antibody (F). Goblet cells were demonstrated by staining with either goat polyclonal antibody to Muc2 (G) followed by staining with Alexa Fluor® 488 donkey anti goat IgG (H+L) or PAS (H; left x 4, right x 40).

The strategy we used was to demonstrate anti-bacterial responses by chick IEC *in-vitro*, and then to ascribe a given activity to an IEC type *in-situ*. We first established primary embryonic epithelial cell cultures obtained from E17 embryos. We deliberately chose embryos prior to internal pipping to avoid previous exposure to microbial elements *in-vivo*. Fig 1 characterizes the morphology of primary epithelial cell cultures obtained from the duodenal loop (Colonies established from other intestinal segments displayed a similar morphology and are not shown). The epithelium spread out from an intestinal fragment (arrow in A) to gradually form a monolayer (A & C). As shown by higher magnification, the monolayer had a typical epithelium-type mosaic structure (B & D). To demonstrate the epithelial identity of these colonies, they were stained for presence of villin and E-cadherin, and as shown, the cells stained positive for both structural proteins (E & F). The presence of goblet cells in the cultures was demonstrated by positive staining to either FITC-labeled goat polyclonal antibodies for Muc2 or to periodic acid–Schiff (PAS) (G & H [2 magnifications]). Negative staining (not shown) was established in macrophage monolayers as well as in fibroblast cultures.

**Fig 2:**
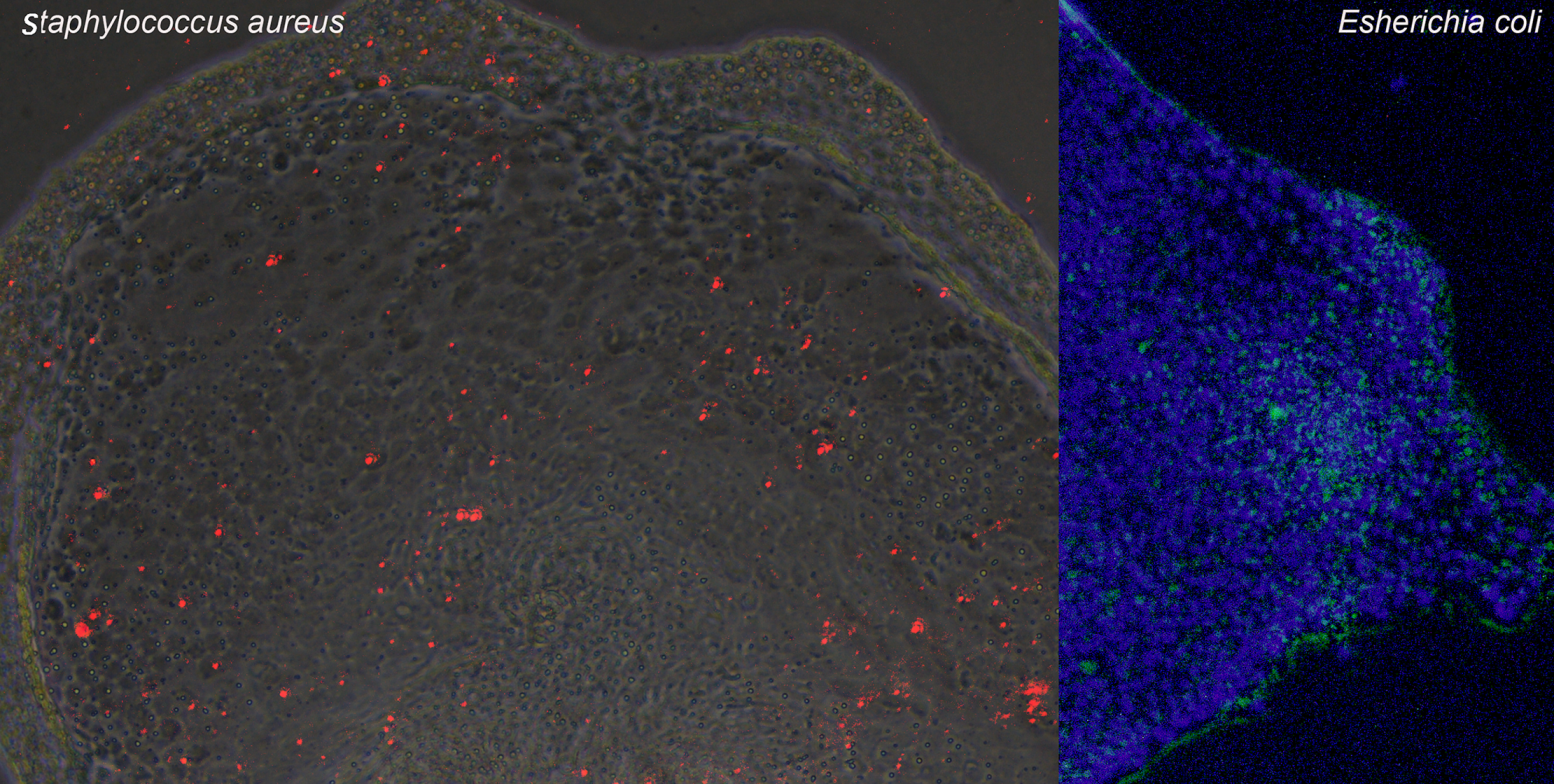
Intestinal epithelial cells internalize and process bacteria conjugated bioparticles. Chick cecal IEC were pulsed with pHrodo™ Green E. Coli BioParticles® or pHrodo™ Red S. aureus BioParticles®. Micrographs were taken after 6 (*S*. *aureus*) or 36 hours (*E*. *coli*).

Next, we tested the ability of IEC cultures to internalize and process bacteria. Cells were cultured and stimulated, as described in methods, with pHrodo™ Green *E*. *Coli* BioParticles® or pHrodo™ Red *S*. *aureus* BioParticles® conjugates; these conjugates do not fluoresce outside the cell at neutral pH but fluoresce brightly when internalized. The results in Fig 2 demonstrate that chicken IEC cell cultures internalized bacteria-conjugate particles. Internalization was time dependent as fluorescing cells increased with time (not shown). Also, cells staining was not homogenous in that the degree of fluorescence differed between positively staining cells.

**Fig 3:**
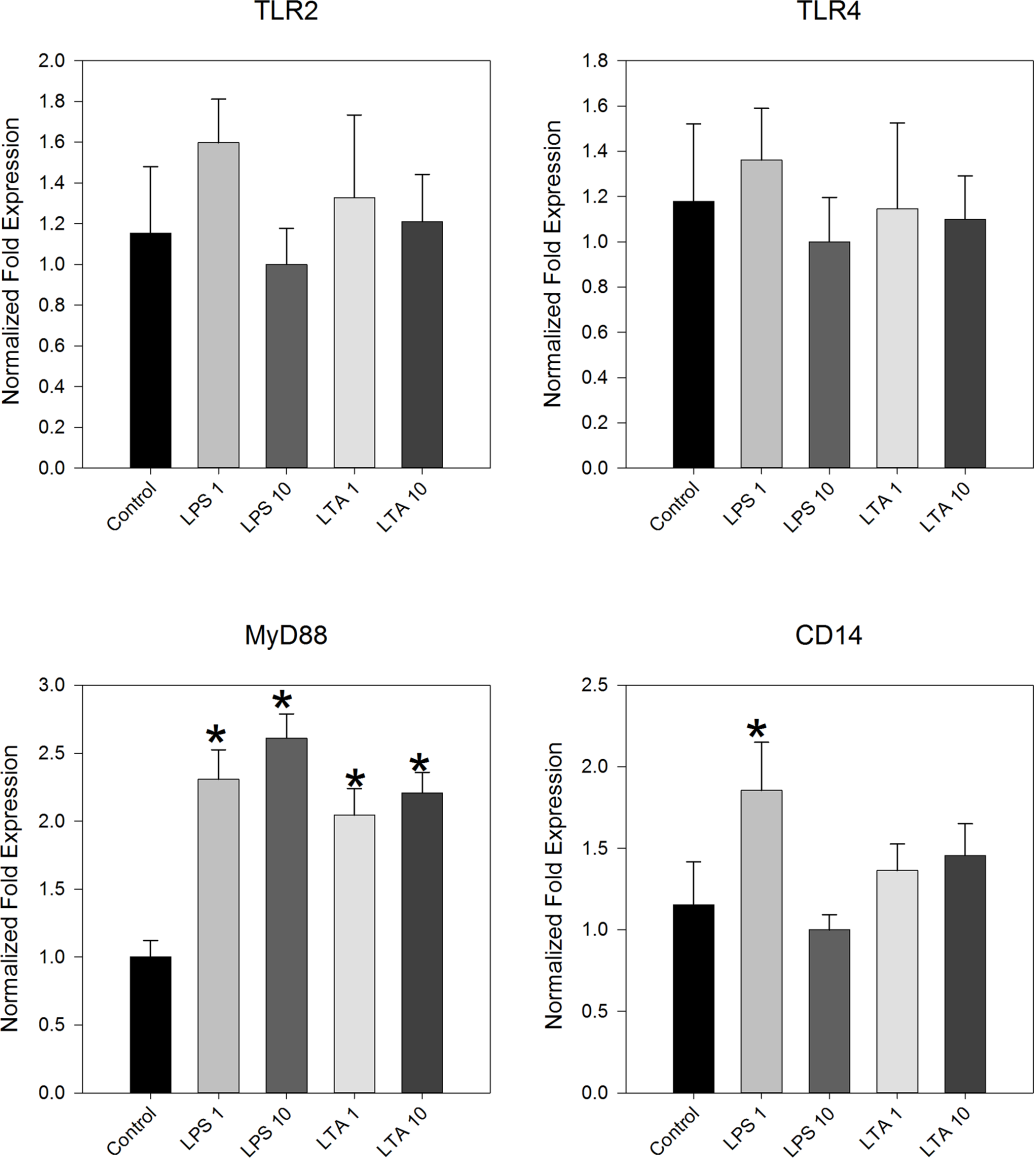
E17-IEC cultures respond to bacterial LPS and LTA. Pooled IEC cultures (n = 20 embryos) were cultured with increasing dosages of *E*. *coli* LPS or *B*. *subtilis* LTA (1 and 10 μg/ml; control = 0) and gene expression was determined 6 hours later (TLR2, TLR4, MyD88 and CD14). Transcriptional levels were determined by quantitative real-time PCR using GAPDH and 18S mRNA as normalizing genes. Results are averages of at least 4 similar experiments ± SEM (* indicates significant responses above control p≤0.05).

As embryonic IEC cultures were shown to internalize bacteria we sought to identify anti-bacterial responses of cultured IEC. In mammalian IECs, binding of PAMPs to their respective PRRs induces a signaling cascade leading to a series of events which include secretion of antimicrobial proteins, secretion of pro-inflammatory cytokines, secretion of chemokines as well as transporting IgA into the intestinal lumen [24]. In order to test if chicken IECs follow the same pattern of responses, we stimulated cultured IECs obtained from the entire intestinal tract (duodenal loop through colon) with bacterial LPS or LTA and tested their responses to the administered stimuli. We initially measured mRNA levels to TLR2 and TLR4 to find indications for induction of a TLR-dependent pathway as well the expression of the co-receptor CD14 and MyD88, a canonical adaptor protein for inflammatory signaling pathways downstream of these TLRs [25]. The results in Fig 3 show neither bacterial product had an effect on the basal expression of TLR2 or TLR4. However, both LPS and LTA stimulation induced a significant increase in MyD88 and less effectively so in expression of CD14 (LPS, 1 μg/ml–P<0.05; LPS, 10 μg/ml–N.S.; LTA, 0.05<P<0.1). Thus, chick IEC cultures respond to bacterial products following a TLR-relevant pathway, without affecting basic TLR expression levels.

**Fig 4:**
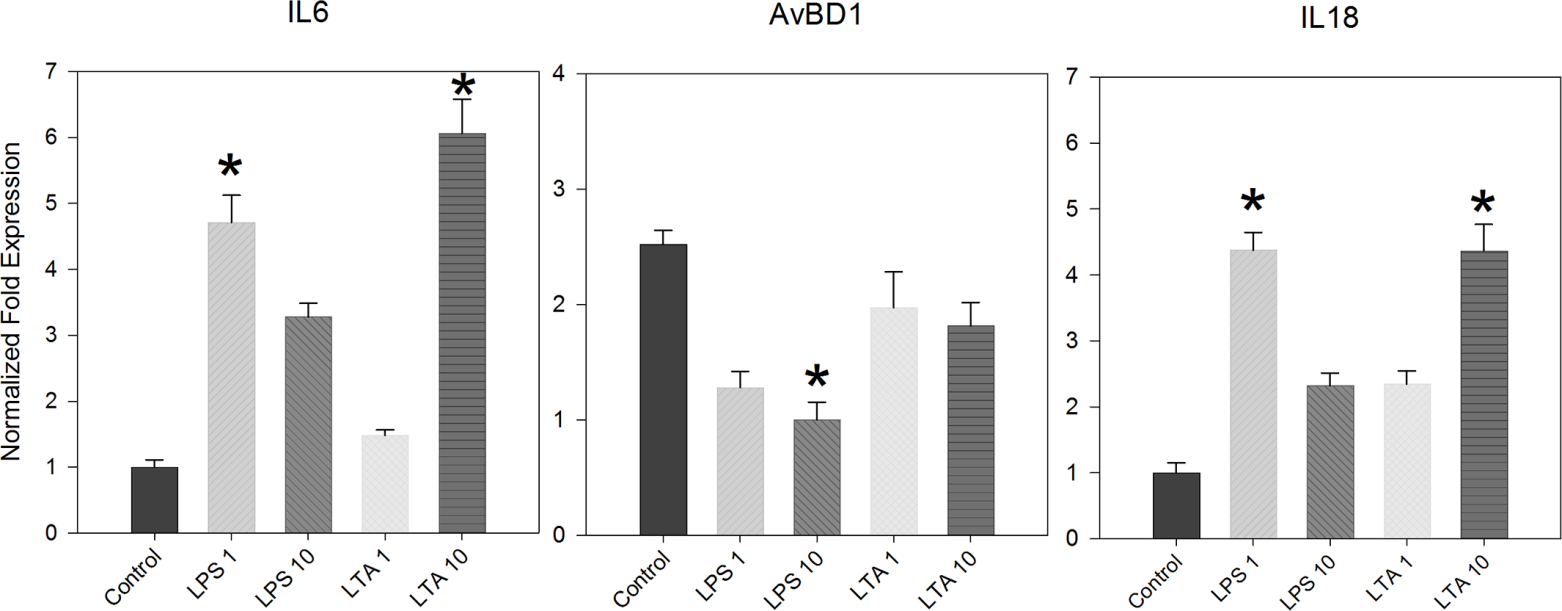
E17-IEC cultures respond to bacterial LPS and LTA. Pooled IEC cultures (n = 20 embryos) were cultured with increasing dosages of *E*. *coli* LPS or *B*. *subtilis* LTA (1 and 10 μg/ml; control = 0) and gene expression was determined 6 hours later (IL-6, IL-18, AvBD1). Transcriptional levels were determined by quantitative real-time PCR using GAPDH and 18S mRNA as normalizing genes. Results are averages of at least 4 similar experiments ± SEM (* indicates significant responses above control p≤0.05).

To extend the previous observations to additional anti-bacterial pro-inflammatory responses, we determined mRNA expression levels of cultured IEC stimulated by LPS and LTA to IL-6, IL-18 and the β defensin AvBD1. Results in Fig 4 show that in vitro stimulation of IEC cultures with 1μg/ml LPS induced a significant increase in the mRNA of the pro-inflammatory cytokines IL-6 and IL-18; 10 μg/ml LPS was less effective. LTA, at 10 μg/ml, induced significant mRNA expression levels of IL-6 and IL-18 (Fig 4). Interestingly, mRNA levels of the β defensin AvBD1 were decreased by LPS and LTA stimulation (significantly so, for LPS at 10 μg/ml).

**Fig 5:**
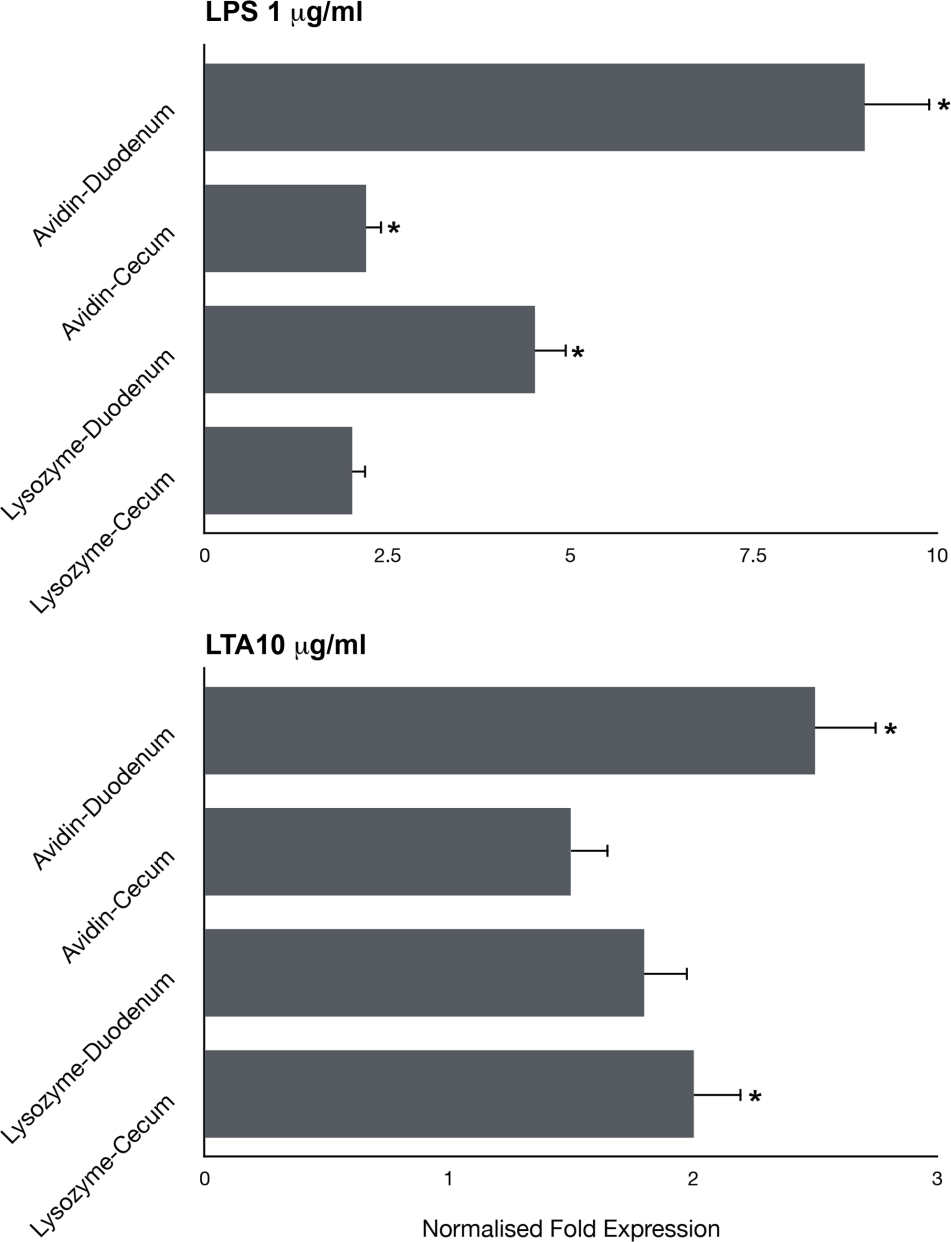
Avidin and lysozyme gene expression following stimulation by bacterial LPS and LTA. Pooled IEC cultures (n = 20 embryos) from either duodenum or cecum were cultured with or without *E*. *coli* LPS (basal and 1 μg/ml) or *B*. *subtilis* LTA (basal and10 μg/ml) and gene expression was determined 6 hours later. Transcriptional levels were determined by quantitative real-time PCR using GAPDH and 18S mRNA as normalizing genes (basal expression was 0.8–1.0). Results are averages of at least 4 similar experiments ± SEM (* indicates significant responses above control p≤0.05).

We then determined effects of bacterial LPS and LTA on the expression of two acute phase proteins, lysozyme and avidin [26–28]. Duodenal IEC cultures were significantly responsive to LPS stimulation as reflected in both avidin and lysozyme mRNA levels (Fig 5): Increased avidin mRNA levels were significant in both duodenal and cecal cultures (P<0.05), while increased lysozyme mRNA levels were only significant in duodenal cultures (P<0.05). The increase of avidin and lysozyme expression in response to LPS stimulation was higher in duodenal cultures. The increase of avidin and lysozyme mRNA expression in response to LTA was of lower magnitude and was similar in duodenal and cecal cultures. Increases were significant for avidin expression in duodenal cultures and for lysozyme expression in cecal cultures (<0.05). Thus, collectively, IEC cultures from embryonic and early post-hatch chicks were capable of internalizing both Gram-positive and negative bacteria and were then able to actively respond to bacterial LPS and LTA as shown by several gene expression assays.

**Fig 6:**
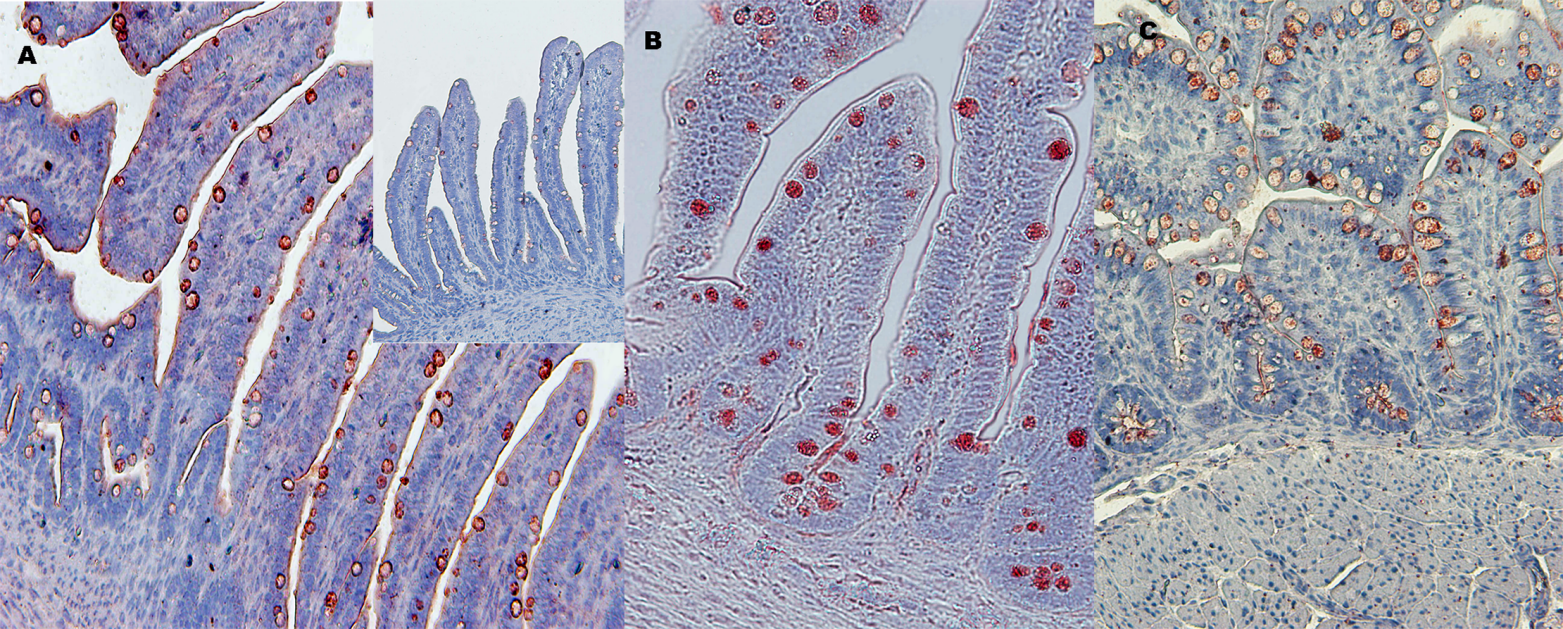
Avidin staining in gut of pre- and post-hatch chicks. Two—3μm thick slides were stained by polyclonal rabbit anti-chicken avidin and color was developed by HRP-labeled goat ant rabbit IgG. A. Duodenum of E19 embryos (x200), B. Cecum of 2-day old chick (x400), C. Colon of 3-day old chick (x400). Insert in A–negative control (only secondary antibody).

**Fig 7:**
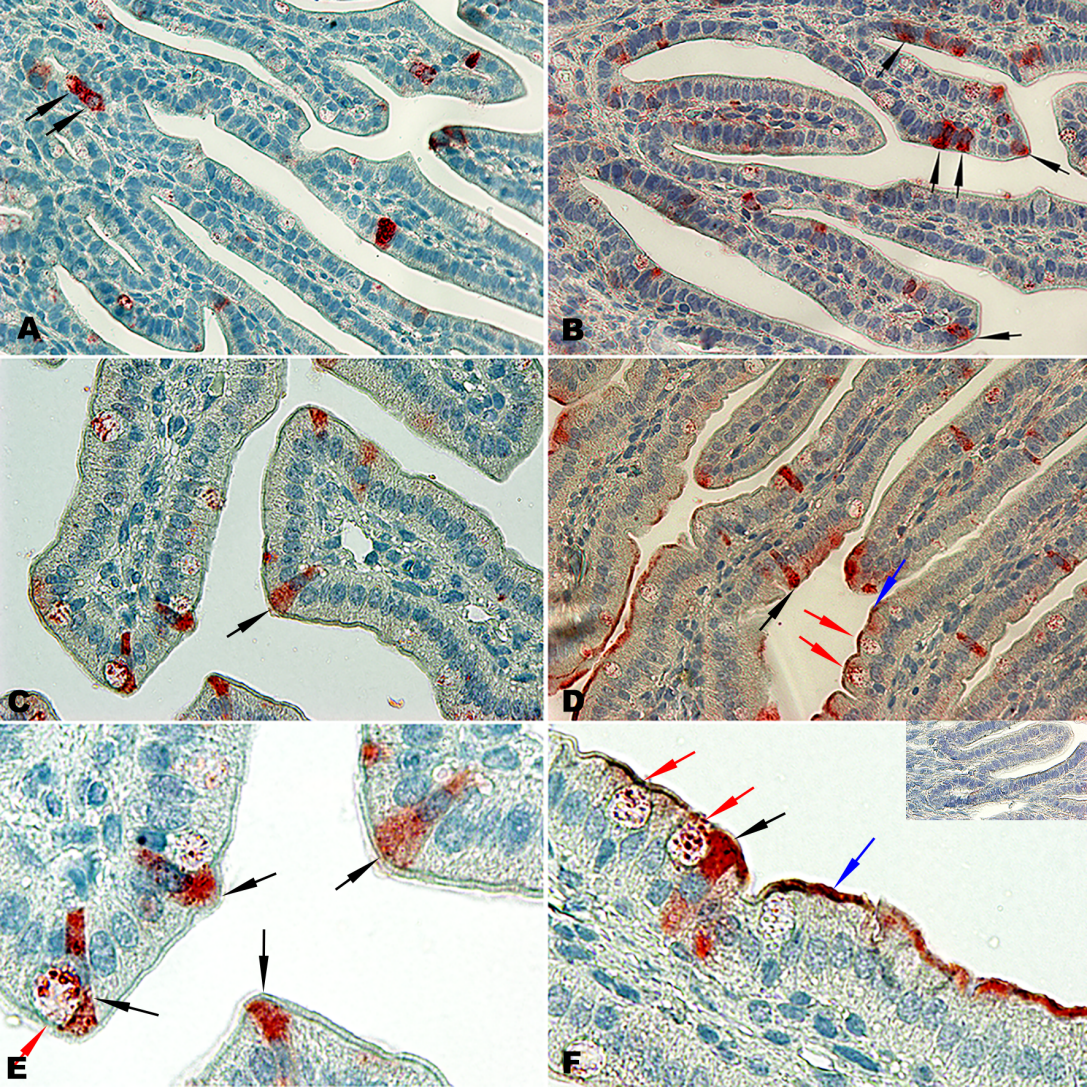
Lysozyme staining in gut of pre- and post-hatch chicks. Two—3μm thick slides were stained by mouse anti-lysozyme and HRP-conjugated goat anti mouse IgG (H+L). A-D duodenum day 0 (x400), E-F duodenum day 0 (x1000). Arrow indications are described in Results. Negative control (secondary antibody only)—Insert in panel F.

As both avidin and lysozyme expression was induced by bacterial products in all IEC cultures, we then localized cells positive for avidin and lysozyme in gut segments derived from pre- and post-hatch chicks (Figs 6 & 7). Avidin-positive cells were observed in all intestinal segments: duodenum, cecum and colon (Fig 6A–6C). The avidin positive cells were almost exclusively identified as goblet cells. Thus, avidin was detected along the entire length of the villus. Avidin positive staining increased with age, with increasing numbers of goblet cells (not shown).

Detection of lysozyme positive cells was of particular interest as it is considered to be a marker for Paneth cells [29], only recently described in the chicken [30]. Surprisingly, lysozyme positive cells were not restricted to the crypts (as in mammals) and positive staining cells were detected at different locations along the villus (Fig 7, micrographs A-F). As expected, lysozyme positive staining was observed in crypts of Lieberkühn (micrograph A, black arrows), however other distinguishable types of lysozyme positive cells were observed along the villus in the duodenum (micrographs B-F): a) Goblet cells (red arrows)–typically defined by their basal nucleus and bloated cytoplasm on the apical side. b) Rod shaped cells (black arrows) defined by their central nucleus and more intense lysozyme-positive staining on the apical side of the cells. Similar lysozyme positive cells were also detected in cecum and colon of pre- and post-hatch chicks (supplemental material). In addition to the lysozyme-positive cell types, we observed that secreted lysozyme remained in a film attached to the apical surface of enterocytes (Fig 7, micrographs D & F, blue arrows). Thus, in the chicken, intense lysozyme staining is not restricted to a single cell type, and the chicken goblet cell appears to be a major role player in anti-bacterial responsiveness.

**Fig 8:**
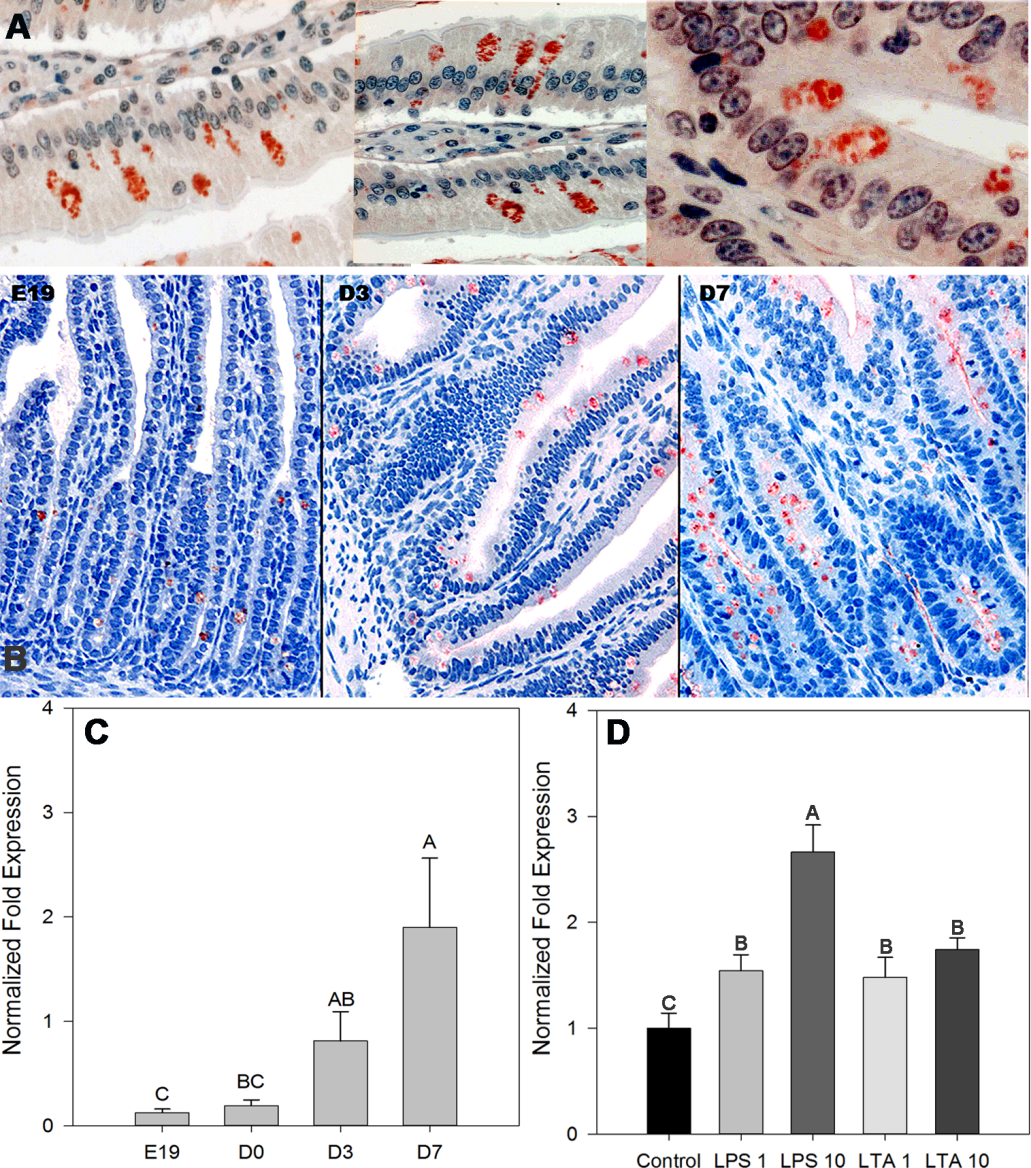
Development of secretory component (GGpIgR) in chick gut. Panel A: Secretory component specific staining in intestine of 30-day old chicks. Two– 3μm slides were stained by mouse monoclonal anti-human secretory component and then HRP-conjugated goat anti mouse antibody (left and central micrographs x200; right micrograph x1000). Panel B: Temporal development of GGpIgR in pre- and post- hatch chicks (Staining–as above) (x200). C. GGpIgR mRNA Expression in pre- and post-hatch IEC. Expression of GGpIgR was determined in RNA prepared from stripped IEC (n = 5 for each time point). Transcriptional levels were determined by quantitative real-time PCR using GAPDH and 18S mRNA as normalizing genes. Results are averages of at least 3 similar experiments ± SEM (Different letters indicate significant differences in GGpIgR expression (P<0.05). D. GGpIgR mRNA expression in E17 IEC primary cultures following stimulation with LPS or LTA. Pooled IEC cultures (n = 20 embryos) were cultured with increasing dosages of *E*. *coli* LPS or *B*. *subtilis* LTA (1 and 10 μg/ml; control = 0) and gene expression was determined 6 hours later. Transcriptional levels were determined by quantitative real-time PCR using GAPDH and 18S mRNA as normalizing genes. Results are averages of at least 4 similar experiments ± SEM (Different letters indicate significant differences in GGpIgR expression (P<0.05).

One other innate antimicrobial activity ascribed to mammalian IEC is to use free secretory component (FSC) to bind luminal bacteria. FSC is formed by cleavage of unbound pIgR at the apical side of enterocytes [31, 32]. To investigate a similar role for the secretory component in the chicken we used secretory component specific immunohistochemistry to study its expression in intestinal tissue; the chicken homologue (GG-pIgR) is bound by anti-human secretory component [33]. Fig 8 (panel A) shows that the receptor was present in chick IEC with marked and intensive staining in goblet cells and the staining was not confined to the baso-lateral surfaces. Expression was very low prior to hatch, and increased dramatically after hatch (Fig 8, panel B). This observation was confirmed, when we used stripped IECs to test temporal expression of GG-pIgR (Fig 8C). Analysis of GGpIgR mRNA temporal expression demonstrated similar dynamics as that were observed using anti-SC antibody, confirming that the antibody recognized the receptor in our tissue slices. To link expression of GG-pIgR directly to anti-bacterial activity we stimulated E17 IEC cultures with either LPS or LTA and determined GGpIgR mRNA expression 6 hours after stimulation. Results in Fig 8D show that stimulation with both LPS and LTA increased GGpIgR mRNA levels (P<0.05).

## Discussion

The functional development of the precocial avian GALT is fundamentally different from that of the altricious mammal [34]; the main differences are in the rate of development as well as in structural organization. Notwithstanding these important differences, immune function along the digestive tract appears to be similar, particularly in terms of innate immune functions. It is now well established that the mammalian gut enterocyte and goblet cell, in addition to their digestive-related functions, are sensors of the luminal environment [35, 36] as well as effective expressers of innate immune functions that serve to protect the intestinal interface [37].

While investigating the immune response-related functions of avian enterocytes and goblet cells, we previously showed that intestinal goblet cells participate in maternal antibody protection as they provide a protective reservoir for maternal IgA antibodies prior to hatch, and in adult chickens they store endogenously-derived IgA [19, 38]. In the present study, we continued to characterize anti-bacterial related functions of the avian gut epithelial cell layer and significantly extend our previous observations by directly demonstrating that chick IEC recognize bacteria or bacterial products, internalize bacteria and respond in different manners to bacterial products. Importantly, we were able to describe the cellular location of three anti-bacterial proteins in all segments of the digestive tract–avidin, lysozyme and the GGpIgR, and that all three proteins were responsive to both Gram-positive and negative stimuli. The three proteins were located predominantly in goblet cells, thus extending the immune relevance of these cells in the chick (discussed below). Of particular interest was the identification of at least three cell types containing lysozyme that were not confined to crypts: the goblet cell, cells positioned in and above the crypts of Lieberkühn, analogous to the location of PCs in mammals, and a lysozyme-positive enterocyte distinctly different in structure and location from that of the classic mammalian PC.

To determine the ability of embryonic epithelial cells to recognize bacterial PAMPs, we analyzed mRNA levels of TLR4 and TLR2 (both are PRRs for LPS and LTA, respectively) as well as the co-receptor CD14 and the downstream MyD88 following stimulation with either LPS or LTA. While no changes were observed in mRNA expression levels of either TLR4 or TLR2, embryonic IEC stimulation with either LPS or LTA induced a diverse pattern of responses, including that of pro-inflammatory cytokines. Both PAMPS induced signaling pathways involving CD14 and MyD88. Previously published data showed that stimulation of epithelial TLRs with PAMPS activates MyD88-dependent signaling pathways that trigger upregulation of pIgR gene transcription and transcytosis, thus offering a functional association between bacterial stimulation of IEC and variety of anti-bacterial responses they control [39].

The avidin and lysozyme response of embryonic IEC cultures to bacterial LPS and LTA led to localization of avidin and lysozyme secreting cells within the epithelial lining. Presence of avidin and lysozyme in mucosal surfaces of oviducts and lungs were previously reported [40, 41]. Presence of avidin secreting cells in mucosal reproductive tissue was previously reported in avian, amphibian and reptilian species [42]. In the domestic fowl, avidin serves as an acute phase protein which is synthesized and secreted by various cells and tissues, during stress, thermal injury or microorganism threats (bacterial or viral) [26, 42, 43]. Using avidin specific immunohistochemistry, we found that avidin was expressed by intestinal goblet cells in chick embryos and post-hatch chicks. The results showing that avidin positive staining was localized to intestinal goblet cells are in agreement with studies showing presence of avidin in oviduct goblet cells [43]. As an acute phase response is a reaction of the organism to local or systemic disturbances in its homeostasis, our observations indicate that intestinal goblet cells participate in gut protection and maintenance of gut homeostasis in embryonic and post-hatch chicks.

Lysozyme is a key effector protein in innate immunity. The protein is abundant in various secretions including saliva, and mucus. It is present in cytoplasmic granules of macrophages and PMNs and is a bio-marker of PC [29]. The antibacterial as well as the immunomodulatory properties of lysozyme which include activation of PRRs and recruitment of leukocytes, were recently reviewed by Ragland and Criss [44]. The presence of lysozyme positive cells was observed not only in small intestine but also in the distal gut segments (colon and cecum) of chick embryos and post hatch chicks. This observation is in line with studies showing presence of PC in colon and appendix of embryonic mammals [45], as well as presence of lysozyme in secretory cells in mucosal tissues [46].

The migratory pattern of PC along crypt-villus axis depends on the expression of EphB receptors and their EphrinB ligands. Both type molecules are surface-bound. Repulsive forces between cells expressing EphB and cells expressing EphrinB affect migration patterns of cells. In mammals, the expression of EphB3 receptor on PC and the expression of EphrinB1 by the differentiated cells in the crypt, are responsible for the downward migration of PC [1, 47]. In contrast to mammals, where lysozyme positive PC migrate downward from the stem cell region where they are formed [45], lysozyme positive cells in the intestinal lining of chickens, were located along the villus and on villi tips post-hatch. Thus, we suggest that in the avian intestinal milieu other factors, yet to be determined, affect the migratory pattern of differentiated cells. When examining the pattern of lysozyme positive cells in the chick duodenum, we noticed in several locations along the villus groups of 2–3 lysozyme positive cells displaying a rod-shaped morphology different from that of the goblet cell. These rod-shaped lysozyme-positive cells have yet to be functionally characterized.

In a recent review by Clevers, studying the intestinal crypt as a stem cell compartment, he mentions the presence and possible function of the “crypt base columnar” cells (CBC) in association with maturing PC [47]. CBC are continuously cycling cells and are considered to be self-renewing multipotent stem cells. While the functional association between CBC and PC has yet to be fully appreciated, the physical association has been demonstrated is several studies [47]. While studying lysozyme-positive cells in the adult chick duodenum, we observed “CBC-like” cell clusters in crypts as well as in higher regions of the villus (S2 Fig.). This observation has led us to hypothesize that the appearance of these cell groups together with the lysozyme-positive rod-shaped cell might indicate that the capacity for cell proliferation and differentiation might occur outside crypts in loci along the intestinal villus in the chicken. This notion is supported by recent findings showing that cell-cell interactions between PC and intestinal stem cells are essential for stem cell proliferation and growth, as PC provide essential signals for their maintenance [48].

The secretory component is a conserved epithelial-derived glycoprotein cleaved from the pIgR receptor that facilitates transfer of IgA from sub-epithelial sites into the intestinal lumen. Studies have shown that about 50% of the pIgR traffics to the apical surface of enterocytes where it is cleaved and released as free secretory component [49]. Free secretory component, via multiple glycosylation sites, displays neutralizing properties against pathogen-associated molecules and acts as an antibacterial substance [50]. The structure of an avian SC was previously described [33], and by applying SC- specific immunohistochemistry we found intensive positive staining in intestinal goblet cells of chicken embryos and post-hatch chicks. Interestingly the staining observed in goblet cells encompassed the entire cytoplasm and was not limited to the basolateral domain. This observation is in line with data showing that the pIgR can be found in both basolateral and apical endosomes in polarized epithelial cells [51]. The granular appearance of the staining may suggest that the avian SC is present in secretory vesicles of goblet cells. This finds support in a recent observation by Xu et al [52] showing that pIgR trafficking may involve both transcytotic and secretory pathways which are distinctively different. These authors proposed a model suggesting different physiological functions for different trafficking populations of pIgR and suggested that sequestration of the SC in specific secretory vesicles may be differently regulated allowing its rapid release upon appropriate stimulation. While staining was minimal in chicken embryos, it increased dramatically post-hatch. This observation was further supported by our observations demonstrating a temporal increase in pIgR mRNA levels in epithelial cells obtained from intestinal tissues of pre- and post-hatch chicks and corresponded with maturation of goblet cells [53], suggesting that the receptor expression was also associated with the functional maturation of goblet cells and the epithelial lining. In vitro stimulation of chicken embryonic IEC with LPS or LTA induced a significant increase in GGpIgR mRNA expression, suggesting that these embryonic cells recognize bacterial PAMPS and are capable responding to their stimulation. Hence, we propose that embryonic goblet cells utilize the GGpIgR for both innate protection, as suggested in this manuscript, and for IgA uptake as we have previously demonstrated [19]. This is supported by studies showing that pIgR can be recycled to both apical or basolateral surfaces of polarized epithelial cells [52, 54].

Finally, we observed that lysozyme, possibly secreted by goblet cells, remained attached to the mucin layer on enterocytes. A similar observation was made in the case of IgA when we previously investigated the behavior of maternal and endogenous IgA in pre- and post-hatch chicks [19]. Thus, collectively, our studies indicate that free SC, SIgA, avidin, lysozyme, as well as other defensive proteins, may become associated with the mucin layer, coat IEC and thereby prevent direct access of bacteria to the epithelial surface.

## Supporting information

S1 Fig Lysozyme positive goblet cells in the distal intestine (colon and cecum) of pre- and post- hatch chicks. Staining details are as described for Fig 7. Micrograph A–Negative staining control, day 2 cecum (x40). Micrograph B–day 2 cecum (x400). Micrograph C–E20 cecum (x400), Micrograph D–day1 colon (x400).
(TIF)

S2 Fig Clusters containing CBC-like cells (H&E) in chick duodenum. Micrograph A is a low-magnification composite of crypt zones containing clusters of narrow dark staining cells imposed between proliferating enterocytes (black arrows; x200). Micrograph B shows similar darkly-staining narrow cell clusters (or single cells) positioned between mature enterocytes in the upper-villus area (blue arrows; x400). Micrographs C-E are high magnifications (x1000) of narrow dark staining cells close to the villus tip (red arrows). C shows a cluster; D shows a rod shaped cell with a narrow cell placed immediately above it (indicated respectively by 2 red arrows); E shows a single cell distinguished from other enterocytes by its narrow profile and dark-purple staining cytoplasm.
(TIF)
