# Supplementary figures and images for: Innate immune functions of avian intestinal epithelial cells: Response to bacterial stimuli and localization of responding cells in the developing avian digestive tract
Source: PLoS One. 2018 Jul 6;13(7):e0200393. doi: 10.1371/journal.pone.0200393 (PMC6034880; doi:10.1371/journal.pone.0200393)

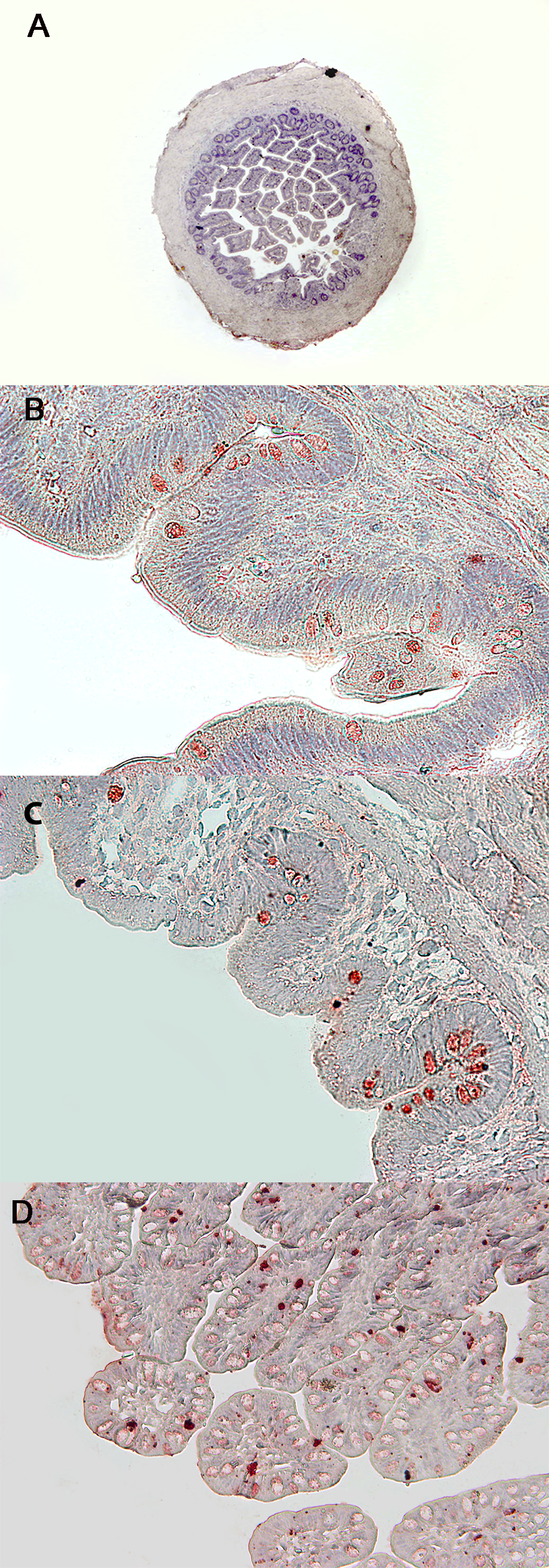

Supplement: S1 Fig — Staining details are as described for Fig 7. Micrograph A–Negative staining control, day 2 cecum (x40). Micrograph B–day 2 cecum (x400). Micrograph C–E20 cecum (x400), Micrograph D–day1 colon (x400). (TIF) [file pone.0200393.s001.tif]

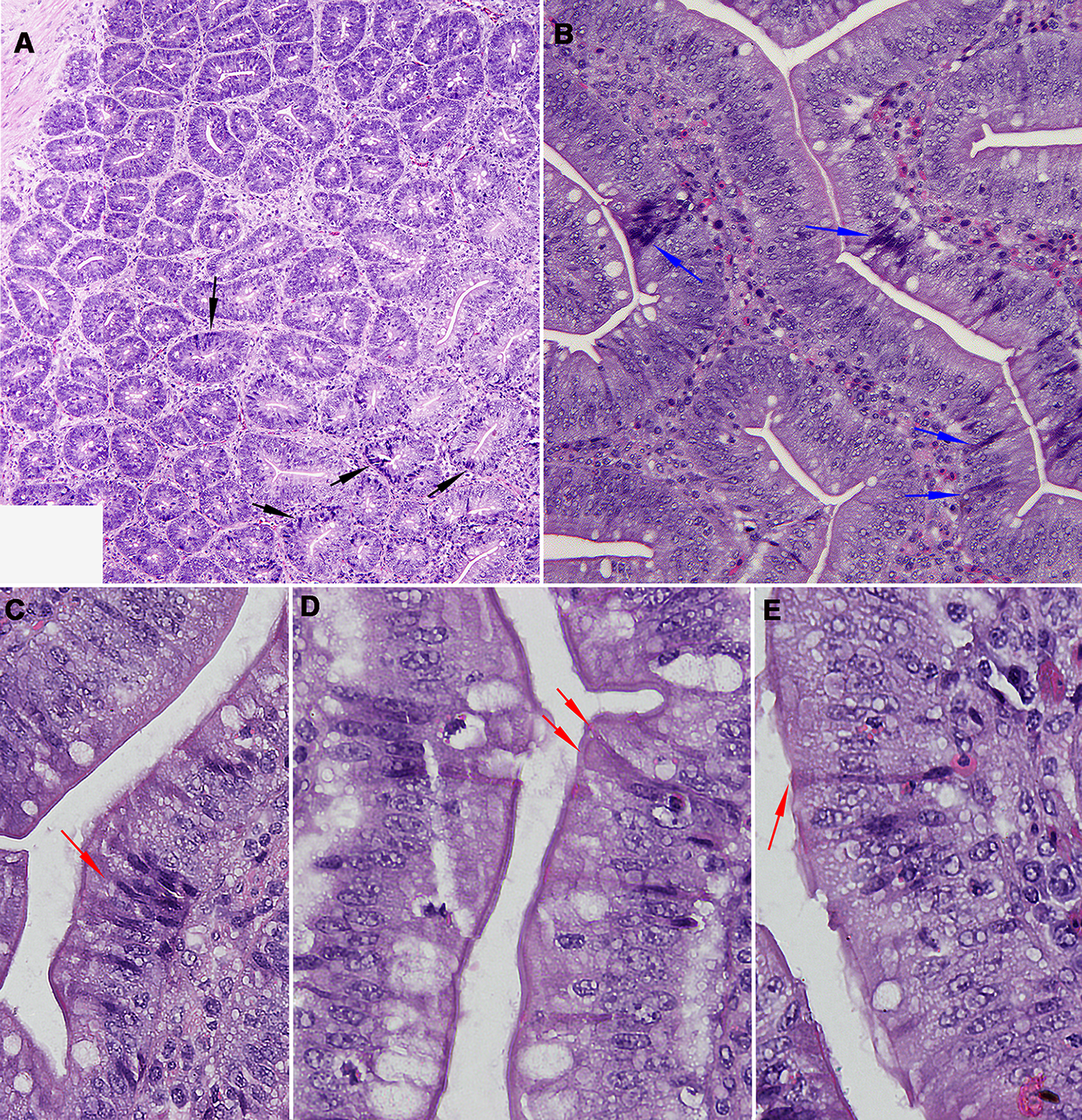

Supplement: S2 Fig — Micrograph A is a low-magnification composite of crypt zones containing clusters of narrow dark staining cells imposed between proliferating enterocytes (black arrows; x200). Micrograph B shows similar darkly-staining narrow cell clusters (or single cells) positioned between mature enterocytes in the upper-villus area (blue arrows; x400). Micrographs C-E are high magnifications (x1000) of narrow dark staining cells close to the villus tip (red arrows). C shows a cluster; D shows a rod shaped cell with a narrow cell placed immediately above it (indicated respectively by 2 red arrows); E shows a single cell distinguished from other enterocytes by its narrow profile and dark-purple staining cytoplasm. (TIF) [file pone.0200393.s002.tif]
